# Food Insecurity Screening in High-Income Countries, Tool Validity, and Implementation: A Scoping Review

**DOI:** 10.3390/nu16111684

**Published:** 2024-05-29

**Authors:** Sabine Baker, Danielle Gallegos, Megan A. Rebuli, Amanda J. Taylor, Ray Mahoney

**Affiliations:** 1 Centre for Childhood Nutrition Research, Faculty of Health, Queensland University of Technology, South Brisbane, QLD 4101, Australia; danielle.gallegos@qut.edu.au (D.G.); a45.taylor@hdr.qut.edu.au (A.J.T.); 2 School of Exercise and Nutrition Sciences, Faculty of Health, Queensland University of Technology, Kelvin Grove, QLD 4059, Australia; 3 CSIRO Health & Biosecurity, Adelaide, SA 5000, Australia; megan.rebuli@csiro.au; 4 Australian e-Health Research Centre, CSIRO Health and Biosecurity, Herston, QLD 4029, Australia; ray.mahoney@csiro.au

**Keywords:** food insecurity, screening, brief tool, COM-B, screening experience, healthcare, implementation

## Abstract

Household food insecurity has significant negative implications across the lifespan. While routine screening is recommended, particularly in healthcare, guidelines are lacking on selection of screening tools and best-practice implementation across different contexts in non-stigmatizing ways. The objective of this scoping review was to synthesize evidence on household food insecurity screening tools, including psychometrics, implementation in a range of settings, and experiences of carrying out screening or being screened. Four electronic databases were searched for studies in English published from 1990 until June 2023. A total of 58 papers were included, 21 of which focused on tool development and validation, and 37 papers described implementation and perceptions of screening. Most papers were from the USA and described screening in healthcare settings. There was a lack of evidence regarding screening in settings utilized by Indigenous people. The two-item Hunger Vital Sign emerged as the most used and most valid tool across settings. While there is minimal discomfort associated with screening, screening rates in practice are still low. Barriers and facilitators of screening were identified at the setting, system, provider, and recipient level and were mapped onto the COM-B model of behavior change. This review identifies practical strategies to optimize screening and disclosure.

## 1. Introduction

Household food insecurity (HFI) is an increasing reality for many families and individuals in high-income countries [1], particularly those on low incomes. HFI has a multitude of short- and long-term negative implications across the lifespan, with particular impacts on health. Among adults, HFI is associated with nutrient inadequacies and obesity, mental illness, and chronic diseases (such as kidney disease, cardiovascular diseases, diabetes, and asthma) [2]. For children and adolescents, HFI has negative impacts on behavior, physical, psycho-social, educational development, and mental health [3,4]. HFI is associated with greater healthcare utilization (primary healthcare as well as acute and emergency care) and significantly higher annual healthcare expenditures [5]. For instance, in Canada, adults had 26%, 41%, and 69% higher odds of acute care admissions when experiencing marginal, moderate, and severe HFI, respectively [6]. In the USA, food-insecure families had 20% greater total healthcare expenditures than food-secure families, for an annual difference of $2456 [7]. Given the significant implications of HFI, there have been calls for monitoring and routine screening to identify at-risk individuals [8]. Currently, food insecurity (FI) monitoring relates to economic access to food, as food budgets are often sacrificed for other household expenses such as housing, utilities (heating and cooling), and medical care. Identifying individuals and families experiencing HFI could be a way to identify those at risk of compromising other social determinants of health.

In the USA, where population-level HFI is monitored annually, several national organizations have recommended routine screening in adult and pediatric health settings, including the Academy of Nutrition and Dietetics [9], the American Academy of Family Physicians, the American Academy of Pediatrics (AAP) [10], American Association of Retired Persons, and Centers for Medicare and Medicaid Innovation [8]. Nevertheless, effective and universal routine screening for HFI remains challenging. More data are needed regarding screening tools and best-practice implementation across different contexts.

A review of US-based validation studies of HFI screening tools in healthcare settings indicated that short tools are being used to identify and refer at-risk individuals to tailored and specialized assistance and resources. The review concluded that screening is, generally, acceptable to patients and clinicians [8]. However, little is known about other settings in which screening might occur, for example, early education and care, school, welfare services, and community organizations.

The goal of screening is to identify individuals and families who may be at risk of experiencing any level of HFI from marginal (anxiety and worry about financial access to food), moderate or low food security (where diet *quality* is compromised), to severe HFI or very low food security (where members of the household have compromised on the *quantity* of food). In any setting, it is important that screening tools have high sensitivity, i.e., accurately detect those who are food insecure as food insecure, and specificity, i.e., accurately rule out those who are not food insecure to ensure constrained resources are effectively allocated. The Hunger Vital Sign™ (HVS), a two-item screening tool derived from the US Household Food Security Survey Module (HFSSM), is being widely used in the USA to screen for HFI.

Calls to introduce routine HFI screening have been met with caution or even concern from patient advocates regarding the potential for screening to cause harm in relation to discrimination, re-traumatization, or legal consequences, such as the removal of children from households [11]. Seeking support for HFI is often accompanied by feelings of embarrassment and stigma or a sense of guilt that resources are being taken from others perceived to be in greater need [12]. As a result, HFI often remains under-reported [13]. This is particularly true for populations who are more at risk of HFI than others and have more at stake (for example, involvement of child protection) if HFI is identified without context. Indigenous populations in high-income colonized countries including Australia (Aboriginal and/or Torres Strait Islander), New Zealand (Māori), Canada (First Nations, Inuit, and Métis), and the USA (American Indian, Native Alaskan, and Native Hawaiian) have well-documented histories of genocide, forcible child removal, and being subject to racist policies, structural violence, and intergenerational trauma. These factors coexist with negative effects due to social determinants of health to place Indigenous populations at significantly higher risk of experiencing HFI [14,15,16].

Successful implementation of universal screening in healthcare, education, and community settings relies on the effective and permanent adoption of changes to routines and systems. Implementation requires the mobilization of physical and psychological resources, as well as behavior change in the key stakeholders (professionals, recipients of services or care, and management) [17]. The Capability, Opportunity, and Motivation Model of Behavior Change (COM-B) has been used extensively to inform the development and implementation of evidence-based interventions [18], such as screening for alcohol use [19], participation in cervical screening [20], chlamydia testing [21], and cardiometabolic screening for people with severe mental illness [22]. It has not, to date, been applied to the implementation of HFI screening. Applying the COM-B model and the associated Theoretical Domains Framework [23] to evidence on the implementation of HFI screening provides a useful framework for synthesizing the evidence to inform practice.

While calls to screen for HFI are gaining traction, little guidance is given about which tool to use and what to consider for efficacious screening implementation. Therefore, this review aimed to synthesize the available literature on HFI screening to inform best practice implementation. Specifically, the study objectives were to (1) identify the tools used to screen for HFI and their validity across contexts and population groups (including Indigenous populations); (2) explore barriers and facilitators for the implementation of HFI screening; and (3) explore the experiences of those doing the screening and being screened. A scoping review methodology was chosen to describe the current research on HFI screening tools. Importantly, the review integrates evidence of tool validity with papers on perceptions of screening and screening implementation in a range of settings, thereby contributing a comprehensive synthesis of food insecurity screening to the field.

## 2. Materials and Methods

This scoping review followed the JBI Reviewer’s Manual for Scoping Reviews and was registered with OSF https://doi.org/10.17605/OSF.IO/FKMJ2, accessed on 2 November 2021. The inclusion criteria, as well as the research questions used, are guided by the PCC (population, concept, and context) mnemonic [24], which is outlined in Table 1.

### 2.1. Search Strategy

Four databases were searched: PubMed, CINAHL, PsycINFO, and Embase. The search strategy was developed in partnership with a research librarian and incorporated keywords related to food insecurity and screening. In addition to the keyword searches, database-specific subject and/or MeSH terms were used in search queries. Search terms and syntax are provided in the Supplementary File. Inclusion and exclusion criteria are listed in Table 2. Due to differences in healthcare, agricultural, and policy systems, the search was limited to high-income countries. Papers were sourced from 1990 until June 2023, were in English only, and were peer-reviewed. Documents that were reviews, books, book chapters, letters, commentaries, government or institutional reports, conference abstracts, or dissertations were excluded. All study designs were included, namely qualitative, cross-sectional, validation, mixed methods, and quality improvement.

### 2.2. Study Selection, Data Extraction, and Analysis

Two authors independently screened titles, abstracts, and full-text articles in Covidence [26] using the eligibility criteria (Table 2). Conflicts were assessed by a third author. Data from each study were independently extracted by two authors. A third author reviewed the extraction tables in Covidence and resolved conflicts. Papers that focused on the development and validation of screening measures were collated. Measure characteristics were narratively synthesized and compiled to provide an overview of tools and to identify gaps in the literature.

Papers that elicited data on screening tool implementation, quality improvement, experiences of screening, or experiences of being screened were reviewed and relevant data were extracted. Qualitative data were initially analyzed using thematic synthesis to inductively identify emerging themes. Coding was conducted by one author (DG), with 11% of articles (*n* = 4) independently coded by a second author (SB). A code book or framework was created inductively, which was developed from line-by-line coding of extracted qualitative data related to the research questions. Each code had a descriptor and relevant quotes were extracted according to the code identified [27]. The code book was then deductively reviewed using the COM-B theoretical framework and factors were mapped onto the six model components [17,18]. The COM-B is an implementation science theory that posits that behavior is a result of the interaction between **capability,** which can be psychological (knowledge) or physical (skills), **opportunity,** both social (societal influences) or physical (environmental resources), and **motivation,** which can be automatic (emotion) or reflective (beliefs, intentions). The COM-B model, alongside the Theoretical Domains Framework, has been previously extensively used in examining clinical behavior change in healthcare settings and was thought to be the most aligned theoretical approach for this review [28].

## 3. Results

**Figure 1:**
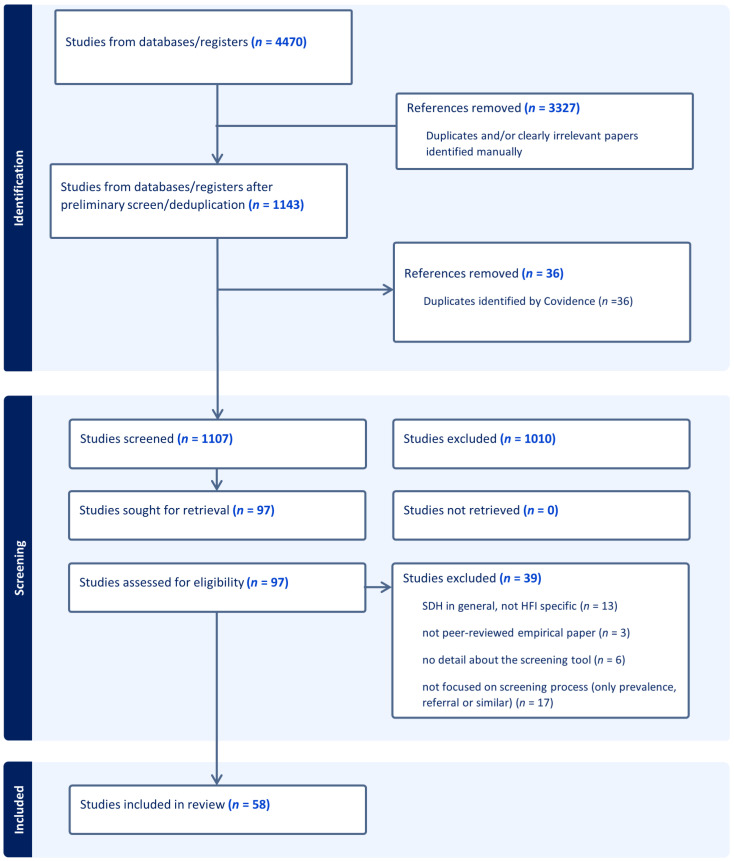
PRISMA diagram.

Database searching identified 4470 initial papers. A preliminary title screen was conducted in EndNote X9.3.1 [29] to remove duplicates and irrelevant papers (e.g., animal, gene, or agricultural studies). Following this, 1143 papers were imported into Covidence. After the removal of remaining duplicates, 1107 papers underwent a title and abstract screen, 97 papers progressed to full-text review, and 58 papers were included in the final review (see Figure 1). In total, 21 papers (36%) described the development and/or validation of screening tools in various settings; 37 papers (64%) described the implementation process and the experiences of those doing the screening or being screened. These two groups of papers were analyzed and described separately.

### 3.1. Overview of Included Studies

Table S3 in the Supplementary File summarizes the papers included in the review. Papers were published from 1997 to 2023 with the majority (59%) published in the last five years. Most (*n* = 50, 86%) studies were undertaken in the United States of America (US), with others from Canada (*n* = 4), Australia (*n* = 3), and the United Kingdom (UK) (*n* = 1). The screening was implemented across diverse settings with most in healthcare (*n* = 50, 86%), of which 40% (*n* = 23) were explicitly in community or primary care sites and one-third (31%) were in hospital settings (*n* = 10; either clinics or emergency departments). Other settings included a community HIV clinic [30], childcare intake [31], dental services [32], and home-delivered meal services in community aged care [33]. Nearly half (47%, *n* = 27) were focused on pediatric populations (predominantly caregivers) and three studies (5%) focused on older adults. No papers described the implementation of screening in Indigenous-specific settings. Only one paper described the validation of screening tools within an Indigenous population, among the Inuit people in the Arctic Circle of Canada [34].

Screening was carried out across different modalities, including self-administered by the recipient (*n* = 21) (e.g., the patient, client, or caregiver) or staff-administered (*n* = 24). Surveys were either completed using pen and paper, a digital format, or a mix of multiple modalities.

#### 3.1.1. Tool Development and Validation

Six tool development/validation papers were primary papers that developed tools by assessing the validity of questions or combinations of questions from current measurement tools [34,35,36,37,38,39]. Two of these used large population census data [34,36], one used a large dataset underpinning the development of the Hunger Vital Sign™ (HVS) [37], and the remaining three used smaller community samples—one in an Australian hospital setting [38], one with Australian pregnant women via an online survey [39], and the other a US community setting [35]. Sixteen papers focused on validating previously determined screening questions, most commonly the HVS™, in different settings with different population groups. Almost all studies used screening questions derived from and validated against the United States Department of Agriculture (USDA) Household Food Security Survey Module (HFSSM).

#### 3.1.2. Screening Implementation and Experiences

Eight papers presented findings from quality improvement projects and interventions that aimed to increase screening rates [40,41,42,43,44,45,46,47]. The remaining 29 papers described the experiences of undertaking screening or being screened. While most (*n* = 13) of these employed qualitative methodologies such as interviews and focus groups, papers with cross-sectional designs (*n* = 11) or mixed methods (*n* = 5) were also included, as were intervention studies (*n* = 2).

### 3.2. Validity of Screening Tools

Table 3 describes the articles focused on FI screening tool development and validation. Most studies (*n* = 17) used the gold standard reference measure for validating the new tools—the USDA HFSSM. The 18-item version was used for households with children and the 10-item version for households without children. The 6-item short form was used in six cases [30,31,32,48,49,50]. Two of these were with households with children [31,32]. One study validated a single question using the HVS [51].

Six articles were primary papers determining the validity of different combinations of questions as a screening tool [34,35,36,37,38,39]. Frongillo et al. [35] concluded that both the Radimer/Cornell and the Community Childhood Hunger Identification Project (CCHIP) questionnaires had good sensitivity and specificity and were valid for the assessment of FI in a general population of rural families with children. Gundersen et al. [36] tested the validity of a two-item screening tool utilizing items from the HFSSM. They concluded that any combinations are acceptable for clinical use and each combination has advantages. The 2-item HVS was validated by Hager et al. [37], which showed that it was sensitive, specific, and valid among low-income US families with young children. Kerz et al. [38] found the 2-item HVS (but phrased as questions instead of statements) to be highly sensitive and specific in an Australian pediatric healthcare setting. McKay et al. [39] found that among pregnant people using an online survey, the combination of two questions related to worrying about running out of money for food and not being able to provide balanced meals was the most sensitive for all households and for households with children. Gundersen et al. [36] and McKay et al. [39] also investigated the validity of two-question screeners for different population groups. Gunderson et al.’s [36] recommended combination of two questions had the lowest sensitivity in the following demographic groups: households with children (97.0%), households where incomes were lower than 200% of the poverty line (97.1%), and where the respondent was over 60 years old at all income levels (96.6%). The specificity of these two questions varied markedly and was lowest in lower-income households with children (<200%—9.5% and <100%—73.7%) but also varied depending on the language spoken at home, ethnicity, and presence of a disability. For McKay et al. [39], the recommended combination of questions was more sensitive for households with low education attainment (90.9%), where women were not living with a spouse (100%) and where household incomes were less than AUD 70,000 (96%). Specificity for different groups did not vary markedly except for households with incomes less than AUD 70,000, where it was 93.1% compared to 98.7% for all households [39]. Urke et al. [34] found that single items could be validly used to rapidly screen for child or adult HFI in Arctic Canada. However, to give a more accurate picture of the food security situation in a family, Urke et al. [34] suggest using a 2-item screener comprised an item relating to adults and an item relating to children. They suggest that basing a rapid HFI assessment on only items drawn from the adult module (as performed in the study by Hager et al. [37]) may not comprehensively capture the household food security situation, particularly with respect to any children present. Results showed that single questions, on average, had lower sensitivity and specificity compared to two or multi-item tools (sensitivity: 59.0–84.9%; specificity 80.0–94.1%) [47,52].

**Table 3:** Summary of tool validity.

Author Year Country/City/State	Name of Screening Tool	Comparator Measure Name (# Items)	Sensitivity % (95%CI)	Specificity % (95%CI)	Validity of Indicators	Summary of Findings
Primary development papers						
Frongillo 1997 US New York State [35]	1. Radimer/ Cornell 2. Community Childhood Hunger Identification Project (CCHIP) 3. NHANES III	Two researchers categorized participants based on interview information including 24 h dietary recall and food stores in the house	Radimer/Cornell 89 CCHIP 86 NHANES III 32	Radimer/ Cornell 63 CCHIP 73 NHANES III 90		Radimer/Cornell and CCHIP had good specificity and excellent sensitivity of the definitive criterion measure. NHANES III item had excellent specificity.
Gundersen 2017 US National [36]	Question combinations from the 18 item HFSSM	HFSSM (18)	Items 1 + 2 97.0–98.7 * Items 2 + 3 96.4–98.7 * Items 1 + 3 98.8–99.8 *	Items 1 + 2 73.7–94.4 * Items 2 + 3 82.5–94.5 * Items 1 + 3 73.8–93.1 *	Accuracy Items 1 + 2 84.1–94.6 * Items 2 + 3 88.6–94.9 * Items 1 + 3 84.8–93.6 *	2-item FI screen can accurately identify HFI. Any combinations have acceptable sensitivity and specificity for widespread clinical use; each combination has advantages.
Hager 2010 US [37]	Question combinations of 1 and 2 questions from the 18 item HFSSM. Based on affirmative responses in food insecure HH two questions selected	HFSSM (18)	Q1 only: 93 Q2 only: 82 Q1 and Q2: 78 Q1 or Q2: 97	Q1 only: 85 Q2 only: 95 Q1 and Q2: 96 Q1 or Q2: 83		2-item FI screen (HVS) was sensitive, specific and valid among low-income families with young children.
Kerz 2021 Australia Brisbane, QLD [38]	Combination of questions from - AHS single question - HFSSM (18) [AUS] - FAO FIES (8) - HVS (2) Based on +ve responses for FI participants—8 questions had +ve responses between 70–90% and were used to create 2-question combinations. 26 combinations were tested	HFSSM (18)	HFSSM 2 + 3: 96.0# HFSSM 3 + 4: 96.0 HFSSM 3 + HVS1: 92.0 HFSSM 3 + FIES3: 92.2 HFSSM 2 + FIES3: 96.0 HFSSM 1 or 2: 96.0 HFSSM 1 and 2: 69.0 HFSSM 2 or FIES3: 96.0 HFSSM 2 and FIES3: 63.0	HFSSM 2 + 3: 90.3 HFSSM 3 + 4: 90.3 HFSSM 3 + HVS 1: 90.1 HFSSM 3 + FIES 3: 95.9 HFSSM 2 + FIES 3: 87.3% HFSSM 1 or 2: 90.3 HFSSM 1 and 2: 100 HFSSM 2 or FIES 3: 87.3 HFSSM 2 and FIES3: 98.0	Tetrachoric correlation using the HFSSM and the potential two-question combinations; HFSSM questions 2 and 3 (r = 0.979) and HFSSM question 2 and FAO-FIES question 3 (r = 0.961).	HFSSM Q2 + 3 has high sensitivity and specificity and may assist practitioners in pediatric healthcare settings in identifying clients who are at risk of FI.
McKay 2022 Australia [39]	2 item screening (various combinations of items from HFSSM)	HFSSM (10)	Q1 + Q2 All HH 82.4% HH with children 77.8% Q2 + Q3 All HH 93.3% HH with children 88.9% Q1 + Q3 All HH 85.7% HH with children 100%	Q1 + Q2 All HH 98.3% HH with children 98.1% Q2 + Q3 All HH 98.3% HH with children 99.1% Q1 + Q3 All HH 99.6% HH with children 98.1%	Accuracy Q1 + Q2 All HH 97.3% HH with children 82.3% Q2 + Q3 All HH 98.0% HH with children 91.1% Q1 + Q3 All HH 98.7% HH with children 99.6%	Q1 + 3 demonstrated best accuracy and reflects both worry about food shortages and cost pressures that have been identified as of concern for food insecure and hungry pregnant women. A 2-item FS screener, created by combining Q1 + 3 of the 10-item USDA tool was found to be sensitive and specific to identify FI pregnant people.
Urke 2014 Canada Arctic Circle [34]	Investigated each item of the 18-item adapted HFSSM	Modified HFSSM 18 item to improve acceptability among Inuit	1. In the last 12 months, were there times when it was not possible to feed the children a healthy meal because there was not enough money? (a) Adult survey 92.3 (b) Child survey sub-sample 88.5 2. In the last 12 months, were there times when you could only feed your children less expensive foods because you were running out of money to buy food? (a) Adult survey 94.8 (b) Child survey sub-sample 96.9 3. In the last 12 months, were there times when the food for you and your family just did not last and there was no money to buy more? 93.0 4. In the last 12 months, did you ever worry whether the food for you and your family would run out before you had enough money to buy more? 91.9	1a. 97.3 1b. 95.4 2a. 81.9 2b. 80.6 3. 93.4 4. 89.1	PPV/NPV/Accuracy 1a. 97.3/92.1/94.7 1b. 95.8/87.3/91.6 2a. 85.0/93.6/88.6 2b. 85.7/95.6/89.5 3. 95.9/88.9/93.2 4. 85.7/95.6/89.5	Screening for child FI #1 best. Adult FI #3 best 1 question from each of the adult and child modules in the 18-item HFSSM can assess FI. Basing a rapid FI assessment on both adult and child items gives a more accurate picture of the FS situation in a family.
**Validation papers**						
Baer 2015 USA Boston MD [53]	HVS	HFSSM (18) for 18–25 yr olds (parents) FSSM (10) for 18–25 yr olds (not parents) FSSM—(9) for youths aged 12–17 used with 15–17 yr olds	88.5	84.1	PPV 72.8 NPV 93.8	Sensitivity lower than expected; specificity comparable. Moderate PPV—patients who screen +ve may benefit from more extensive questioning to determine presence and severity of FI
Bayoumi 2021 Canada Toronto [51]	Single FI item from NutriSTEP “I have difficulty buying food I want to feed my child because food is expensive”	HVS (2)	84.9 (72.4, 93.3)	91.2 (89.4, 92.8)	False +ve 8.8 (7.2, 10.8) PPV 31.3 (26.7, 36.2), NPV 99.2 (98.5, 99.6) NLR 0.2 (0.1, 0.3) PLR 9.6 (7.7. 12.0) Accuracy 90.9 (89.1, 92.5)	Single NutriSTEP FI question may be an effective screening tool in clinical practice to identify MFS in families with young children.
Crichton US [54]	HVS	Community-level FI statistics (USDA Food Access Research Atlas) based on individual residential zip codes	Q1 only 21 Q2 only 23 Q1 or Q2 25	Q1 only 88 Q2 only 83 Q1 or Q2 80	PPV Q1 67 Q2 60 Q1 or Q2 58 NPV Q1 only 49 Q2 only 49 Q1 or Q2 50 Accuracy Q1 only 52 Q2 only 51 Q1 or Q2 51	Discordance between HVS and the USDA’s food access atlas data, not confident in the ability of the screening tool to accurately detect food security in population with trauma.
Gattu 2019 US [55]	HVS	HFSSM (18)	96.7	86.2	PPV 65.7 NPV 99	The HVS identifies children at risk of FI in primary care and ED.
Harle 2023 US [48]	Epic EHR food insecurity screener (HVS as questions)	HFSSM (6)	94.5 (91.2- 96.8)	93.1 (90.6- 95.2)	AUC 0.938 95% CI 0.921- 0.955	EHR-based FI screening was accurate compared to single-domain screener.
Harrison 2021 US [56]	HFSSM-2 (HVS)	HFSSM (18)	Q1 only: 92 (94–100) Q2 only: 88 (80–97) Q1 + Q2: 83 (72–93) Q1 or Q2: 98 (94–100)	Q1 only: 95 (92–98) Q2 only: 95 (92–97) Q1 + Q2: 98 (97–100) Q1 or Q2: 91 (87–94)	PPV Q1 only 80 (70–90) Q2 only 78 (67–89) Q1 + Q2 93 (86–100) Q1 or Q2 70 (59–80) NPV Q1 only 98 (97–100) Q2 only 97 (95–99) Q1 + Q2 96 (94–99) Q1 or Q2–99 (98–100)	HFSS-2–high sensitivity, specificity, PPV, NPV–supports the use of HFSS-2 for adults in the general medical population.
Kleinman 2007 US Chelsea MA [52]	Single Q: Hunger Screening Tool “In the past month, was there any day when you or anyone in your family went hungry because you did not have enough money for food?”	HFSSM (18)	83	80	77% time to time reliability (kappa= 0.54) cf HFSSM time to time reliability of 83% (kappa = 0.66)	Single-question screening tool can identify family hunger as part of routine healthcare in a primary care pediatric clinic serving a low-income community.
Lane 2014 US Baltimore MD [47]	Single Q: “In the last year, did you worry that your food would run out before you got money or food stamps to buy more?”	HFSSM (18)	59	87	PPV 70 NPV 81 PLR 4.5 NLR 0.47 Stability kappa 0.69 substantial agreement between clinic and lab based administered tools	Single question screen in a primary care setting can effectively identify families with FI.
Makelarski 2017 US Chicago Ill [49]	12-month HVS and AAP 30-day HVS and AAP	HFSSM (6)	12 month: AAP 76 (65, 85) HVS 94 (86, 98) AAP (HH with children) 78 (61, 90) AAP (HH no children) 71 (52, 86) HVS same for both groups: 94 (81, 99) 30 day AAP 72 (56, 84) HVS 91 (79, 98) AAP (HH with children) 67 (46, 83) AAP (HH no children) 79 (49, 95) HVS (HH with children) 93 (76, 99) HVS (HH no children 100 (78, 100) 12-month recall study AAP tool admin. after HFSS and the HVS 67 (46, 83) AAP tool admin. before HFSS and HVS 79 (54, 94)	12 month: AAP 93 (85, 97) HVS 82 (72, 90) 30 days: AAP 96 (88, 99) HVS 83 (73, 91)	12 month: AAP NLR 0.3 (0.2, 0.4) HVS NLR 0.1 (0.0, 0.2) AAP PLR 11 (5, 23) HVS 5 (3, 8) Of those who screened negative with the AAP tool but positive with the HVS tool, 92% screened positive because they selected “sometimes true” for 1 or both HVS survey items 30 day AAP NLR 0.3 (0.2, 0.5) HVS NLR 0.1 (0.0, 0.3) AAP PLR 17 (16, 53) HVS PLR 5 (3, 9) Of those who were missed by the AAP tool but captured by the HVS, 90% screened positive because they selected “sometimes true” for 1 or both HVS survey items	In an urban population with a high prevalence of FI the 12-month and 30-day recall versions of the tool recommended by AAP lacked sensitivity, HVS 12-month and 30-day recall versions were highly sensitive in this population. Both tools, 12-month recall version was more sensitive and at least as specific as the 30-day recall.
Radandt 2018 US Seattle WA [32]	HVS	HFSSM (6)	95.4	83.5		The 2-item FI screen was found to be sensitive and reasonably specific, to identify FI families in a pediatric dentistry clinic.
Swindle 2013 US [31]	Two questions of the six item HFSSM	HFSSM (6)	78.6	97.4	Internal consistency α = 0.82	A 2-item screen for FI conducted by childcare providers was valid.
Tran 2022 US [57]	NutriSTEP FS question “I have difficulty buying food I want to feed my child because food is expensive”	HVS (2) USDA HFSSM (18) Canada HFSS (18)	NutriSTEP v. HVS—67.7% v. HFSSM 82.1% v. Canadian HFSS 67.6% HVS v. HFSSM 92.9%	NutriSTEP v. HVS 87.1% v. HFSSM 94.1% v. Canadian HFSS 92.9% HVS v. Canadian HFSS 85.3%	FI NutriSTEP/HFSSM 92% PPV FI NutriSTEP/Canada HFSS 92% PPV FI NutriSTEP/HVS 84% PPV FS NutriSTEP/HFSSM 86.5% NPV FS NutriSTEP/Canada HFSS 70.3% NPV FS NutriSTEP/HVS 73% NPV FI HVS/HFSSM 83.9% PPV FI HVS/Canada HFSS 93.5% PPV FS HVS/HFSSM 93.5% NPV FS HVS/Canada HFSS 83.9% NPV	NutriSTEP FS question had good validity in identifying FI cf. USDA HFSSM. HVS yielded high sensitivity and specificity, cf. Canada HFSS. HVS over-detected FI, NutriSTEP under-detected FI cf. USDA HFSSM. Sensitivity and specificity of NutriSTEP FS question were in the good and excellent range cf USDA HFSSM, indicating the NutriSTEP adequately assessed FI.
Vaudin US [33]	Expanded Food Security Screener (FSS-Exp)	Follow up visit	NS	NS	NS	The final FSS-Exp tool has the potential to be used in healthcare settings to identify community-dwelling older adults who are in need of nutritional support.
Vest 2021 US [50]	HFI Screening question from EHR (HVS) and ICD-10 Z codes	HFSSM (6)	HVS 73.0 (55.9, 86.2) ICD Z score 100 (96.4, 100)		HVS PPV 50 (27.2, 72.8) AUC 0.698 (0.555, 0.841) *p* < 0.05 ICD Z score PPV 100 (29.2, 100) AUC 0.523 (0.487, 0.550)	The two screening approaches did not perform well overall in this sample of safety-net patients. ICD-10 Z codes underestimated prevalence of social risk factors and are limited in their potential to effectively infer the presence of a social factor for a patient. Social risk factor screening questions were very specific but had higher sensitivity than ICD-10 Z codes. The screening questions performed better than ICD-10 Z codes but did not reach the level of being diagnostically useful. The combination of screening questions and ICD-10 Z codes resulted in small improvements in performance.
Young Australia Sydney NSW [30]	Two questions of the HFSSM (6)	HFSSM (6)	100 (75, 100)	78 (61, 90)	NPV: 100 (88, 100) Internal reliability 2-item α = 0.94 6-item α = 0.90 Correlation Co-efficient between two tools: ρ = 0.895; 95% CI 0.821, 0.940; *p* < 0.0001) κ agreement of responses between the two questionnaires was 0.650 (*p* < 0.0001).	The two-item FS is a valid, reliable, and sensitive tool for clinical use in people living with HIV to identify FI.

NS = Not specified; USDA = United States Department of Agriculture; AAP = American Academy of Pediatrics; CCHIP = Childhood Community Hunger Identification Project (8-Item); EC-FM = The Early Childhood Family Map Inventory (2-Item from HFSSM); FAO-FIES = Food and Agricultural Organization Food Insecurity Experience Scale (8-Item); FSS-Exp = Expanded Food Security Screener (10-Item expanded version of HFSSM 6-Item); HFSSM (3-Item) = USDA Household Food Security Survey Module (3-Item); HFSSM (6-Item) = USDA Household Food Security Survey Module: 6-Item Short Form; HFFSM (18-Item) = USDA Household Food Security Survey Module: 18-Item; HVS = Hunger Vital Sign™ (Hager) (2-Item); NHANES III = Third National Health and Nutrition Examination Survey; NHS = Australian National Health Survey (1-Item); NutriSTEP = Nutrition Screening Tool for Every Preschooler (1-Item); HH = Households; PPV = Positive Predictive Value; NPV = Negative Predictive Value; AUC = Area Under Curve; CI = Confidence Interval; PLR = Positive Likelihood Ratio; NLR = Negative Likelihood Ratio; ICD = International Classification of Diseases. * range across US population and high-risk demographic groups.

Ten papers reported on the Positive Predictive Value (PPV) or Negative Predictive Value (NPV) [30,34,47,50,51,53,54,55,56,57]. The PPV was lower for the HVS in safety-net patients (PPV 50) [50], when respondents were adolescents and youths aged 12–25 years (PPV 72.8) [53]; for caregivers of children < 4 years [55]; and for single item questions (NutriSTEP PPV 31.3 [51]; “worry question” PPV 70 [47]). However, Tran and Bellini [57] suggested that while a single-item screener (NutriSTEP) under-detected FI compared to the HFSSM, it still adequately assessed food insecurity (PPV 92). Harrison et al. [56] identified improvements in PPV when both questions of the HVS were asked, rising from an average of 79 for each single item from the HVS (Q1 or Q2) to 93 for both HVS questions. NPVs for tools with multiple questions were largely similar ranging from 83.9 to 100. Lower NPVs were for single items [47,57]. The lowest NPV of 49 was for the predictive value of the HVS with the USDA’s food access atlas data [54].

Three papers explored convergent validity [37,53,56]. In the development of the original HVS, Hager et al. [37] showed attenuated but statistically significant child health outcomes using the HVS and the HFSSM. Harrison et al. [56] demonstrated convergence between the two-item screener and the HFSSM for adults with diabetes, heart disease, and asthma, but not for depression. Baer et al. [53] demonstrated attenuated but statistically significant outcomes between FI and social problems between the one question and the HVS.

While screening questions were administered in a number of different modalities, such as on paper and electronically, no study considered the impact of modality on tool validity.

#### Wording of Tools

The screener most often used was the two-question Hunger Vital Sign™ (HVS; see Box 1). In the literature, this is also referred to as the Hager tool, HFSSM-2, or the AAP-recommended tool. It should be noted that the AAP-recommended tool uses the same two questions but with a dichotomized answering option. Screeners with answering options limited to yes/no responses tend to be less sensitive than ‘often’, ‘sometimes’, and ‘never true’ response options (76% vs. 94%) but more specific (94% vs. 82%) [49].

When tested in Australian hospital pediatric outpatient clinics, items two and three of the HFSSM (referred to as the HVS), phrased as questions rather than statements, had a sensitivity of 96% and a specificity of 90% [38]. The same tool using the original statements, when tested in US pediatric populations in hospital emergency departments and clinics, had sensitivity and specificity of 97% and 83%, respectively [38]. Variations in the timeframe of the tools from 30 days to 12 months were also tested. In an urban population with a high prevalence of food insecurity, the 12-month recall version of both the HVS and the AAP tool was more sensitive and at least as specific as the 30-day recall [49].

Box 1 **Hunger Vital Sign™.**

    1. Within the past 12 months, we worried whether our food would run out before we got money to buy more.
    2. Within the past 12 months, the food we bought just didn’t last and we didn’t have money to get more.

Response options:

     ● often true
     ● sometimes true
     ● never true
     ● don’t know/refused

A positive screen is indicated by answering ‘often true’ or ‘sometimes true’ to either question.

Australian wording:

    1. Within the past 12 months, have you ever worried that food will run out before you are able to buy more?
    2. Within the past 12 months, have you run out of food and not had enough money to buy more?

### 3.3. Perceptions of Screening Tools and Tool Implementation

**Figure 2:**
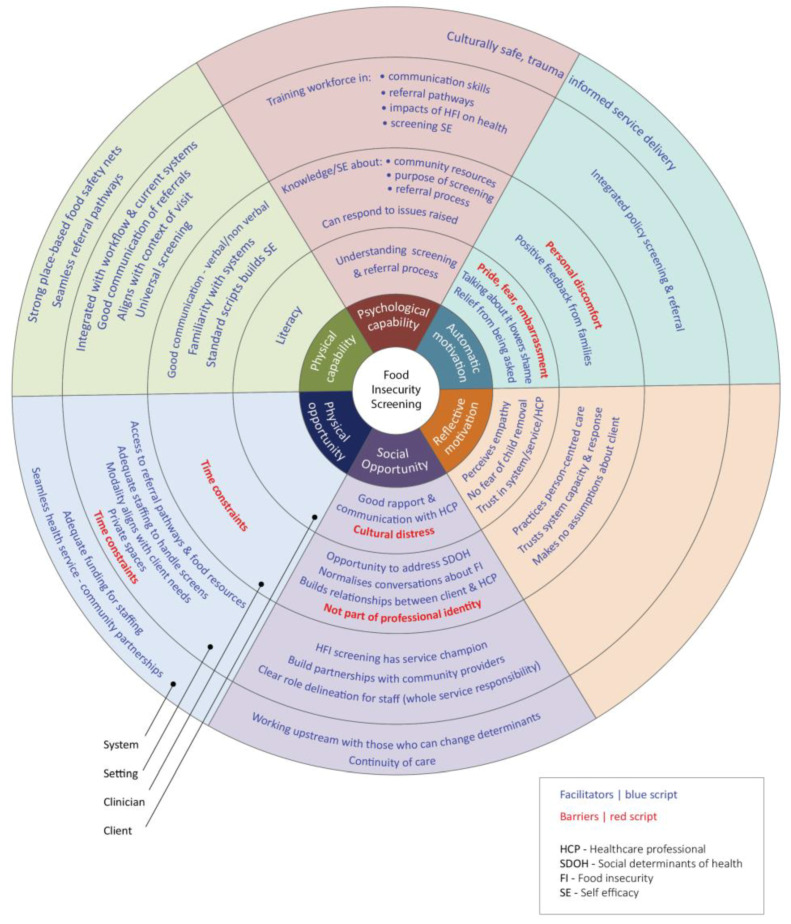
Barriers and facilitators to FI screening at recipient, provider, setting, and system level mapped onto COM-B components.

In addition to considerations around which screening questions to use, the review of papers identified factors (barriers and facilitators) that influence screening implementation. These were identified as falling under four categories: client/recipient, clinician/provider, setting/organizational, and system-level factors. These factors were mapped onto the COM-B model and are highlighted in Figure 2: physical capability (e.g., lack of interpersonal skills), psychological capability (e.g., lack of knowledge about referral pathways), reflective motivation (e.g., beliefs regarding the relevance of screening), automatic motivation (e.g., embarrassment and shame), physical opportunity (e.g., mode of testing, time constraints), and social opportunity (e.g., stigma). Given that most papers describe screening in healthcare settings, the figure shows barriers and facilitators for this setting.

#### 3.3.1. Recipient/Patient/Client Factors

The effectiveness of screening revolved around the patient’s/client’s level of comfort with screening and/or willingness to disclose their FI status. Typically, those who were FI were less comfortable with screening [13,58].

Clients expressed difficulty in disclosing FI because they felt undeserving of help and thought that other families or patients could use FI resources more.

*“I don’t want to put ‘yes’ on [the FI screener] knowing there’s probably people who can’t even get their kid one package of bacon. I feel like those resources should go to those people, but I don’t know what you do about the people in the middle who don’t qualify for SNAP [Supplemental Nutrition Assistance Program] but aren’t quite able to get what they need…”*

[59] (p. 4)

The recipients of the screening clearly identified the importance of clinician interaction with respect to HFI screening. There were perceptions that clinicians were “just not interested” and that they could be judgmental and lack empathy [60,61,62,63,64,65,66].

*“…there was a doctor who made me feel very uncomfortable, and like she didn’t care about my concerns…She just blatantly said that there were people in society that had worse problems than I did, and I should not be so emotional about it…”*

[66] (p. 600)

Culturally responsive interactions were highlighted as necessary to readily facilitate the exchange of information and to normalize conversations around FI. This was especially important given the level of shame and stigma attached to FI and fears of child removal if FI was disclosed [61,62,63,67,68].

*“Your heart skips a beat when your doctor asks. You automatically go to oh, my God. Somebody’s going to try and take my kids”*

[63] (p. 3)

Recipients indicated they never raised the issue of HFI as it was never raised by the clinician [69]. Once it was raised, the feelings of shame were alleviated and there was a sense of relief that their health professionals were aware of the situation [57,62,64].

*“I love [my pediatrician]. He wasn’t judgmental or raise his eyebrows. He made me feel okay with it and said a lot of people are experiencing FI”*

[62] (p. 5)

From the recipient’s perspective, they were more likely to disclose FI if they either had a close relationship with the screener or if the process was completely impersonal [58,63,64].

*“I feel comfortable with the social worker. They’re there to help you and make sure everything’s okay and they’re less intimidating I think than maybe a doctor or nurse…”*

[59] (p. 5)

Disclosure was also enhanced if there was privacy, no children were present, and if the reasons for the screening and referral responses were fully explained so that fears regarding child removal were alleviated [58,59,62,63,65,70]. Those responding that they were food insecure indicated they wanted referrals and information on resources but that connections to resources needed to be optimized. Financial assistance including vouchers and coupons was preferred over food.

#### 3.3.2. Clinician/Provider Factors

Several clinician-related factors have been identified including who should screen and the important role clinicians play in ensuring screening occurs at a systems and setting level. At an individual clinician level, the main identified barriers to implementation center around their perceptions regarding how clients would respond to screening [60,62,66], fears of alienating their patients by being intrusive [71], their desire to ensure that individuals were not stigmatized including maintaining their privacy, and protecting children [62,66]. This was compounded by their own awkwardness, their perceived lack of capability to ask the questions sensitively, and not being able to respond appropriately to the potential “Pandora’s Box” of social determinants that could arise [13,61,62,72]. Healthcare providers often have limited knowledge of food insecurity and its link to health conditions or disorders [73].

*“It’s a really personal question. You’re asking about money…it’s awkward”*

[72] (p. 4)

*“[I did not screen patients], if I did not have a relationship with them; if patients were not mine, I did not feel comfortable”*

[65] (p. 344)

Clinicians were also reluctant to screen if they thought there would be unmet referrals, the system would not be able to manage the number of positive screens, and they were unaware of the resources they could recommend [13,44,45,60,67,70,71,72,74].

*“I just feel like once you ask about food insecurity, I feel like from there, it will… There may be other needs. ‘Okay, then here’s this food pantry.’ And then it’s like, ‘Yeah, I understand the food pantry is there, but I don’t know how to get there,’ or ‘I don’t have internet.’ I feel like there needs to be someone, like a case manager, being able to provide other supports and services as well”*

[75] (p. 6)

Clinicians also lacked capacity to screen (e.g., [65,72,73]). Some of this lack of capacity was recognized as systems issues addressed below but perceived lack of time was also noted as an important barrier to screening [68].

*“There’s a lot of other things to talk about during the visit, and [there’s] just not the time to identify social determinants”—Healthcare worker*

[68] (p. 90)

Clinician-related facilitators for effective screening included building on and facilitating open communication and trust with patients; this included interpersonal skills such as being able to build rapport, demonstrate empathy, and use effective verbal and non-verbal skills. Universal screening with standard scripts took the guesswork out of who should be screened and how and normalized conversations around FI [13,45,60,61,62,66,72].

Screening increased the awareness of FI as a barrier to treatment [45]. Additional benefits identified by clinicians included that HFI screening provided an opportunity to have a conversation about the social determinants of health, it acted as a potential opportunity for the patient to bring up other issues, and it resulted in more tailored advice [13,64].

*“…it wasn’t just a yes or no answer… the patient proceeded to then tell me more about what their experience [with food insecurity] was like”*

[64] (p. 259)

*“I explained how we can give them [families] suggestions to access healthy food and cheaper options. I also asked them how they were balancing their funds to access food; so, it would be a lot of back and forth”*

[65] (p. 345)

Food insecurity screening was seen as the best practice for patient care, which could reduce readmissions and result in improved patient outcomes [76].

*“I thought it was really high yield because it was something I hadn’t particularly asked about before, and it was nice to have an exact script to use, and then it really got into something that I felt like I could help the family with”*

[60] (p. 26)

#### 3.3.3. Setting Factors

The review identified practical and setting considerations that revolved around how and where screening would be undertaken and by whom. The screening was self-administered (by the patients/recipients themselves in waiting areas and in individual consultations) or clinician/staff administered by administrative staff (e.g., office manager) [75] or by medical, nursing, and allied health staff (e.g., [43]). For example, in the context of HFI screening in United States children’s hospitals, screening was most frequently carried out in inpatient units (58.8%), with social workers (35.5%) and nurses (34.2%) conducting the screening most frequently [77]. The modality was either verbally in-person, using pen and paper, electronically, or a combination (see Table S3 in the Supplementary File). While it was generally seen as advantageous to have one person assigned to carry out the initial screening, there were also benefits to having multiple staff/clinicians engaged in the process.

*“It’s helpful to have multiple people who are responsible for asking this because it establishes that as a culture that this is an important part of healthcare”*

[75] (p. 3)

How screening occurred was linked to privacy and efficiency, with integration into electronic medical records described as the most consistent and efficient [43,58,62,63,72,78,79]. Several papers indicated that patients were more likely to disclose FI if the screen was self-administered [44,58,65,80] or completed in writing [81] as the experience was perceived as non-threatening and with enhanced privacy.

*“Caretaker comfort levels and disclosure of social risk are higher with tablet-based screening”*

[79] (p. 5)

Screening rates varied greatly by setting, provider, patient characteristics, and mode of encounter (e.g., in-person vs. telehealth). For example, while food insecurity screening in initial primary healthcare encounters overall appeared low, the rate of screening in US community health centers with a shared electronic health record was higher during in-person encounters (9.2%) compared with 5.1% at telehealth encounters [82]. A cross-sectional survey of the United States children’s hospitals identified that the majority (67.1%) of pediatric hospitals screened at least some individuals for FI. About one-third of institutions universally screened for HFI on admission [77].

To ensure culturally safe and trauma-informed interactions, other factors in the setting included ensuring that there was a private space in which screening could take place [61,70]. This privacy included ensuring disclosure was not undertaken in front of others or in the case of adults with children, not in front of children given that they may be unaware of the household’s food situation. However, this was difficult to achieve in crowded rooms and facilities with limited space.

*“Unfortunately, it’s (screening) not very private. And what I mean by that is that it’s open to more medical assistants that are sitting at that station and potentially another patient getting vitals next to them…So as they’re asking them the questions, there are more people around and it’s not very private. Sometimes we do have that response of no, no, no I’m fine and then they get inside and they tell the doctor maybe something different”*—Clinician

[83] (p. 10)

Screening was more likely to occur if relevant staff who were best placed to carry it out were identified and screening was incorporated as part of their role description. The identified staff member was context-specific and related to the clinical model of care, resourcing, and timing [41].

There was evidence that screening for HFI was not an appropriate use of time for emergency physicians or specialists [13] and is not the remit of doctors [74]. For example, about a third of participants with Cystic Fibrosis or carers of children with Cystic Fibrosis felt that physicians had limited understanding of FI or should not be burdened with non-medical issues [59].

*“[Physicians] should be more concerned about the problems that are going on, the things that can be done to help health-wise”*

[59], (p. 5)

For in-person encounters, perceptions of power and the relationship between screening provider and recipient are important considerations and in health settings, this often precluded medical staff [13,71]. However, in other settings, medical staff were preferred [78].

*“I feel comfortable with the social worker. They’re there to help you and make sure everything’s okay and they’re less intimidating I think than maybe a doctor or nurse”*

[59], (p. 5)

Outside of the health system, screening was undertaken by child-care and aged-care staff. For screening to be successful, each setting needs a champion and leadership on the ground communicating with organization decision makers about the necessity of prioritizing HFI screening [13,67,74].

*“It’s really convincing the employer that this is necessary and making the argument to the hospital system or to the business that this is necessary”*—Female physician, private practice, 16 years

[74] (p. 15)

Opportunistic screening during health professional–client contact for other purposes was also discussed. In primary care, two papers advised that HFI screening should not take place at well-visit appointments where other screening occurs, such as screening for pediatric developmental issues [72,74]. Screening was regularly carried out in the emergency department. While it was noted that screening in this setting was difficult when people are acutely unwell and some studies excluded those who were critically ill [37,49,55,81], others described successful integration of screening [13,81]. Screening at routine primary healthcare visits could be more desirable given these are regular and longitudinal and enable a wide range of topics to be discussed [60]. Undertaking screening in waiting areas increased efficiency by facilitating more time in consultations to discuss solutions [70]. Deployment was setting-specific and there was no consensus on best practices. Screening needs to be context-specific and dependent on the population being served [62].

*“We’ve got endless numbers of short, categorical screening questions that we tend to check off on checklists and do a very poor job of actually counselling people and their problems. This just adds to the number of those sorts of things that none of us ever has enough time to deal with adequately. A lot of these things we screen for, at the root of it are serious economic, social, and family problems”*

[74] (p. 14)

#### 3.3.4. Systems Factors

Three themes were identified under systems factors acting as both barriers and facilitators. These included clinical flow and integration, referral pathways, and access to community-based resources.

There was general recognition that incorporating HFI screening questions into electronic medical records (eMR) improved integration into the clinical throughput. This ensured that the standardized screening was universally undertaken and that it was not forgotten by healthcare professionals [13,40,41,42,60,64,70,72,78,84]. The inclusion of HFI screening into the eMR also provided an opportunity to automate the system, including algorithms to prompt triage follow-up, provide feedback and cues for action, and facilitate referrals for assistance [70]. Automation also ensured that all clients, irrespective of whether they had previously screened as food secure, were rescreened, given the cyclical nature of FI [64]. There were some reservations regarding integration into the eMR related to the bureaucracy attached to incorporating a new tool [42], the complexity of the software programs [62], the complexity required to track referrals and link to outcomes [84], and the associated costs [67]. Questions also remain regarding where HFI is documented and which staff have access to the information [60]. Related to the integration into eMR was the concomitant language based on ICD10, which has not always had a specific code for FI [41].

One of the key factors for successful HFI screening was ensuring that referral pathways and access to community-based resources were established and integrated into the system [13,40,60,61,62,64,68,69,70,74,84]. There was also recognition that access to resources needed to be widely available in communal areas for those who were food insecure but did not disclose [69].

*“Combining structured screening with broad resource and referral availability for all families may be a promising approach”*

[69] (p. 1490)

One of the most significant barriers at a systems level was a lack of resources to undertake screening. This is primarily related to the lack of staff to screen or to follow up and the lack of time due to the pressure of patient throughput [13,62,64,65,67,70,72,74,85]. In some instances, the lack of time with individual patients means that questions and the issues followed up need to be prioritized [62,67]. The automation of referrals and the integration into eMR as well as clinician training would, in some respects, ameliorate these pressures.

*“We are tremendously understaffed in terms of social services. We have one social worker for a clinic that has >15,000 visits per year. This fact seems overlooked”*

[13] (p. 53)

*“At first it was like, ‘oh my, another question.’ But truly, it wasn’t that much additional time”*

[72] (p. 5)

## 4. Discussion

This is the first comprehensive review of HFI screening combining both tool validity and implementation practices. The included papers showcased a range of tools implemented across health and community settings using several modalities. However, there was inconsistency in reporting across studies on the tool name and framing of the included questions and there was also inconsistent reporting of psychometric properties of HFI screening tools, with few papers including comprehensive data on reliability and validity. Findings relating to best practice implementation suggest that routine health checks provide a good opportunity for screening to occur, though it is essential that screening is undertaken in a way that is sensitive to the needs of individuals and is non-stigmatizing. To optimize HFI screening disclosure, barriers relating to staff resources, knowledge of referral pathways, and lack of access to assistance particularly need to be addressed.

### 4.1. Screening Tool Selection

Screening tools need to be selected based on several factors, including the time it takes to administer the tool, alongside its validity. The gold standard for FI screening is the 18-item USDA HFSSM; however, this is time-consuming to administer. Short screening tools exist that perform well compared to the 18-item HFSSM, most notably, the commonly used 2-item HVS, a shortened version of the HFSSM. There was a lack of consistency when referring to the HVS. In different studies, the same questions were referred to as the AAP tool, the Hager tool, and items 1 and 2 (or 2 and 3) of HFSSM; in some cases, the statements were framed as questions or with different answering options. Despite this, the 2-item HVS screener appears to be the screener of choice in most settings with acceptable sensitivity and specificity. Single-item questions, including the individual questions of the HVS on their own, tend to lack sensitivity and specificity and may not be useful unless they are developed and validated for a specific context and purpose [51,52,57].

The two items in the HVS encompass a ‘worry about food’ question, typically used to identify marginal FI, and a ‘running out for food’ question, used to identify severe FI or very low food security. Research indicates detrimental outcomes for individuals and families who are marginally FI, such as poor educational outcomes and emotional and behavioral problems in children, as well as maternal major depression and anxiety [86,87].

The accepted protocol for assessing HFI using the USDA HFSSM is to group marginally food-secure with food-secure households [88]. This recognizes that those households who have indicated concern have not yet changed food consumption practices but may already be undertaking income or consumption smoothing [89]. However, sociodemographic characteristics of households reporting marginal food security are more similar to those experiencing more severe FI than food secure households [86,87]. Increasingly, marginal FI is being reported separately and this was highlighted in the current review, where studies using the HFSSM as a reference measure used different scoring impacting the sensitivity, specificity, and results of validity testing.

When aiming to screen households for FI risk, excellent sensitivity is more important than specificity to ensure that families who experience FI are correctly identified. The HVS, with an average sensitivity of 93.3% (88.5–95%), misses only 7% of potentially food insecure households. The lower end of this range was for young people (<25 years of age), suggesting that the HVS may lose some sensitivity for younger age groups [52]. The average specificity of the HVS (83%) indicates that 17% of families who may be food secure are falsely identified as at risk of FI. Employing a tool with lower specificity for screening may not be detrimental; however, this does have implications for resource-constrained settings.

The choice of screening tool and the scoring system employed needs to take into consideration context-specific factors. For example, settings in areas with many households experiencing high levels of disadvantage may be willing to absorb low specificity to use the screen as an opportunity to start a conversation about other social determinants of health. Areas with a high level of households that are more advantaged may choose to require positive responses to both HVS questions to identify FI, which increases specificity to 96% but potentially decreases sensitivity to 78% [37].

The wording of the screening tool questions needs to be appropriate for the context. For example, Kerz et al. [38] showed that changing the US framing of the HVS items from statements to questions for Australian use improved the validity of the tool. Response options also matter, for example, families worried about stigma or negative consequences of disclosure may be more likely to endorse ‘sometimes’ than yes/no responses [49]. While brevity is a key consideration in screening tools, sensitivity should not be compromised, and dichotomized answering options seem to achieve lower sensitivity [49]. Future research should investigate the utility of including an item that asks about the children’s food situation for programs that specifically target child FI.

The screening modality has been considered in the context of maximizing tool completion and disclosure rates (e.g., [58,79,80]). Self-administration or administration of the questions by a trusted professional seems to maximize disclosure and minimize discomfort [90]. However, administration may impact tool validity. Future research should investigate the impact of screening modality on both validity and acceptability. The four practical considerations for screening tool implementation are outlined in Table 4.

Currently, there is a clear dominance of screening in health services, compared to other settings, with healthcare providers directly addressing the negative health consequences of food insecurity. However, low-income individuals and families who are at higher risk of food insecurity may face greater barriers to accessing healthcare in the first place due to the lack of accessibility and affordability of services. Many FI households also make decisions to forego healthcare in favor of food [91]. Consequently, there are potentially missed opportunities for integrating screening beyond healthcare including screening in community and education settings. Screening in these contexts may be more likely to reach those at the highest risk of food insecurity and contribute to destigmatizing and normalizing conversations about HFI.

The review uncovered no information on the implementation of HFI screening in Indigenous-specific settings. The impacts of colonization could potentially amplify the barriers to reporting HFI, such as beliefs and fears around child removal, and perceptions of surveillance rather than assistance. Research is needed to examine the validity of tools for use with Indigenous people and the perceptions of HFI screening with Indigenous peoples.

There is also little information on the validity, efficacy, and acceptance of FI screening for those with lived refugee experience, who speak languages other than English, or who come from diverse cultural backgrounds. While there are some data available on Spanish-speaking groups [70,78,81], many of the included studies from English-speaking countries excluded non-English speakers.

### 4.2. Systematic Implementation of HFI Screening

This review used the COM-B to explore the factors that would most impact screening implementation and perceptions of providers and recipients. Figure 2 maps out and provides details of these qualitative findings. These are summarized into four areas that need to be addressed to ensure efficacious HFI screening in any setting.

1. *Ensure that screening is embedded in service delivery that is person-centered, culturally safe, and trauma-informed*

FI is a deeply personal and stigmatizing state with profound impacts on physical and mental health [92,93]. Many individuals experiencing severe FI are currently, or have previously, experienced trauma such as adverse childhood experiences, domestic and family violence, racism, and structural violence [94]. This, in turn, generates a lack of trust in relationships with others, including service providers. Service provision, irrespective of the context and setting, needs to be person-centered, culturally safe, and trauma-aware [95]. Education and training on working in a trauma-informed culturally safe way increase provider buy-in, capabilities (including highly developed interpersonal skills and building strong trustworthy client relationships), confidence, and, ultimately, screening rates (reflective motivation). Such an environment would mean screening is undertaken in a way that is sensitive to the needs of individuals and is non-stigmatizing. This could mean universal screening, consideration of who undertakes the screening, who is present at the screening, and how the screening is undertaken and where.

2. *Orientate staff towards FI as a modifiable determinant of health, wellbeing, and development*

The failure to implement screening is linked to a lack of provider knowledge and skills, combined with negative perceptions. Those implementing the screening are more likely to engage in the activity if they feel confident in why it is being implemented and how they will respond. Knowledge and skills acquisition are required regarding the determinants and sequelae of FI, how to ask follow-up questions sensitively (psychological capability), and understanding the potential fear, stigma, and shame in disclosing FI status (automatic/reflective motivation). Providers need practical training (physical capability) on how and when to screen and the resources and referral pathways available. Familiarity with the systems also facilitates action. Until it is fully socialized to the context, clinicians may need scripts that are in line with the Dreyfus Model of Skill Acquisition (rules to develop competency and importance of training) [96].

3. *Provide systems-level and organizational structures to facilitate universal screening*

Setting and system factors are important to create physical and social opportunities, for example, adequate resources, organizational commitment, and training for screening implementation. While no optimal combination of screening and referral activities exists to date, carefully planned implementation of HFI screening into processes and quality improvement cycles can dramatically increase screening rates. Once integrated into a single streamlined workflow and if supported by continuing education, screening can be sustainable and does not noticeably increase the overall workload. Successful implementation across unique contexts and settings relies on program adaptability and trialability. Common elements for success include organizational policies, on-site leadership and championing, and clear role delineation.

4. *Build organizational partnerships and pathways for referral to address immediate food needs and longer-term determinants of FI*

Successful implementation of routine HFI screening seems more likely when there are referral pathways or resources to alleviate the problem. As most of the studies occurred in the US, the focus was on referral to federal programs (which are not necessarily available in other countries). Notably, there was recognition that not all families/patients were eligible and that local resources needed to be identified and mobilized. Pathways need to be available for eligible and non-eligible families and information about resources needs to be available to everyone if an individual does not want to disclose. Increasingly, health services are also exploring opportunities for providing food safety nets through the development of community-health service partnerships. Where no federal programs or established referral pathways to resources exist, providers often seemed hesitant to screen. However, even in the absence of referral pathways, it is crucial for services to assess the prevalence of the issue to correctly identify the need for assistance, to create ‘pull-demand’, and consequently have data to advocate for better resources.

### 4.3. Strengths and Limitations

This review is the first comprehensive review of food insecurity screening. Other existing reviews focus on the US context only or limit screening to specific settings, such as primary care. The current review includes a comprehensive analysis of both qualitative and quantitative articles and takes into consideration both the practical aspects of screening (choice of tools and delivery modalities) and the impact of screening on the recipients. By employing the COM-B model and identifying specific key recommendations, the review aims to inform practice.

Some limitations need to be considered. The review was limited by restricting the search to peer-reviewed articles in English. Due to the chosen methodology, studies that screened for HFI in the context of specific health conditions may have been excluded if the study did not specifically focus on the process of screening. Despite our relatively broad inclusion criteria, most included studies were small observational studies, carried out in a US context.

In addition, we did not include studies if HFI screening was part of broader social determinants of health screening to ensure results were specific to FI. However, examining the implementation of HFI screening in the context of screening for a range of additional social determinants of health could yield different recommendations.

Given that this review was exploratory in nature, critical appraisal of studies was not conducted and some included papers may have been of poor quality or not comprehensive (e.g., research briefs).

## 5. Conclusions

Overall, the extant literature indicates that valid and reliable tools exist that can be employed to screen for HFI by various professions in a variety of settings across diverse populations. No modality of screening was found to be preferable over another; however, successful implementation was more likely when screening was universal, aligned with client preferences, and ensured confidentiality and culturally safe and trauma-informed approaches. For successful implementation, attention needs to be paid to upskilling staff regarding the importance of addressing FI and building skills in asking about and managing FI. Organizations need to make a commitment to implementation including integrating screening into workflows, providing leadership, and undertaking continuous quality improvement. Finally, services need to build community partnerships that minimize the siloing of service delivery and optimize access to an immediate food safety net as well as to services to address the underlying determinants of FI. More attention needs to be paid to screening in educational or other community settings. The lack of literature and evidence on the development and implementation of screening for Indigenous populations must be addressed.

While there is wide agreement that screening is beneficial and best practice at least in healthcare settings, screening rates appear to remain low in both clinical and community settings. This review identifies practical strategies that can be implemented to close this gap.

## Figures and Tables

**Table 1:** Population, concept, and context (PCC) criteria.

Population	Participants being screened for (clients/recipients) or undertaking the screening (clinicians, healthcare workers, professionals, teachers, early education and care providers) for HFI (across the life span)
Concept	Screening for HFI
Context	High-income countries in any relevant setting including healthcare and community settings

**Table 2:** Inclusion and exclusion criteria for the scoping review.

Inclusion	Exclusion
High-income countries as indicated by World Bank criteria [25]	Low- and middle-income countries
Across the lifespan (including children/youth)	Involves screening tools for diagnostic criteria or symptomatology that do not focus on screening for food insecurity
Utilizes a screening instrument with the intent to specifically assess household food (in)security (main focus of the study is screening/identifying people who experience food insecurity)	Interventions that target food insecurity (unless they include screening) Generic social determinants of health screening unless HFI is reported separately
Involves screening or case finding in any setting in which screening for health may occur (e.g., tertiary, secondary, and primary healthcare or, more broadly, in community settings, schools, etc.)	
Reports on the type of screening tool used	
Reports on the experiences of screening or being screened for food insecurity	Reports on prevalence rates only
Reports on quality improvement projects or workforce development to implement food insecurity screening	

**Table 4:** Practical considerations for screening tool implementation.

1.	Choose a screening tool that is fit-for-purpose and has high specificity and sensitivity for the population to be screened
2.	In most settings, use a minimum of two questions, with at least one question capturing marginal FI and one capturing severe FI
3.	Undertake universal screening, ensure that all users of a service are screened to reduce stigma
4.	Choose a modality that works for both the users of the service (e.g., literacy, English language, and privacy needs) and for the workflow of the organization

## Data Availability

The data supporting this Scoping Review are from previously reported studies and datasets, which have been cited. The processed data are available from the corresponding author upon request.

## Supplementary Materials

The following supporting information can be downloaded at https://www.mdpi.com/article/10.3390/nu16111684/s1: Table S1: Search terms for key review concepts; Table S2: Exemplar database search strategy for PubMed. Table S3: Overview and description of included papers.
