# Supplementary material for: Food Insecurity Screening in High-Income Countries, Tool Validity, and Implementation: A Scoping Review
Source: Nutrients. 2024 May 29;16(11):1684. doi: 10.3390/nu16111684 (PMC11174716; doi:10.3390/nu16111684)
Supplement: Supplementary file 1 [file nutrients-16-01684-s001.zip › nutrients-2995121-supplementary.pdf]

Supplementary Materials

Title: Food insecurity screening in high income countries, tool validity and implementation: A scoping review

Table S1. Search terms for key review concepts

Food security	Screening
“food insecur*” OR “food secur*” OR "food shortage" OR "food scarcity" OR “Food supply” OR “food poverty" OR "food insufficiency" OR hunger OR “hidden hunger” OR “food assistance” OR “food access” OR “food stress” OR “food hardship” OR “food deprivation” OR “household food availability”	“screen*” OR “mass screening” OR “Hunger Vital Sign”

Table S2. Exemplar database search strategy for PubMed

Search number	Syntax
1	"food secur*"[Title/Abstract] OR "food insecur*"[Title/Abstract] OR "food shortage"[Title/Abstract] OR "food scarcity"[Title/Abstract] OR "food poverty"[Title/Abstract] OR "food stress"[Title/Abstract] OR "food hardship"[Title/Abstract] OR "food insufficiency"[Title/Abstract] OR "food access"[Title/Abstract] OR "household food availability"[Title/Abstract] OR "food assistance"[Title/Abstract]
2	"hunger"[Title]
3	S1 OR S2
4	"food assistance"[MeSH Terms] OR "food deprivation"[MeSH Terms] OR "food supply"[MeSH Terms]
5	S3 OR S4
6	"screen*"[Title/Abstract] OR "hunger vital sign"[Title/Abstract]
7	"mass screening"[MeSH Terms]
8	S6 OR S7
9	S5 AND S8
10	#5 AND #8 Filters: Humans, English

Table S3: Overview and description of included papers

Author, Year, Country	Design/ Methods	Aim	Screening Tool	Modality	eMR	Setting	Participants (N; % female; age)	Prevalence of Food Insecurity (%)
**Development and validation papers**								
Baer, 2015, USA	XS	Assess HVS compared with the HFSSM in an urban youth primary care clinic.	HVS vs. HFSSM (18-item)	Self-administered, digital	NS	Hospital	Patients (400; 69; 15-25 yrs)	HVS: 39.5; HFSSM: 32.5
Bayoumi, 2021, Canada	XS	Examine the diagnostic test properties of the FI item taken from NutriSTEP questionnaire.	NutriSTEP item vs. HVS	Staff-administered, NFS	NS	Community - Paediatric Practices (TARGet Kids! Cohort)	Children (via caregiver) (1174; 47; aged 24-50 months)	NutriSTEP: 3.4; HVS: 4.5
Crichton, 2022, USA	XS	Determine FI prevalence in hospitalized trauma patients and mechanisms by which providers can easily identify FI	HVS	Staff-administered, NFS	NS	Hospital - Trauma Centre	Patients (136; 38; $\bar{x}$ 46.2 yrs)	33.8
Frongillo, 1997, USA	XS	Assess the validity of questionnaire-based measures in identifying households with hunger and FI	Radimer/ Cornell vs. CCHIP vs. NHANES III	Staff-administered, NFS	NA	Households	Women (189; 100; 18-39 yrs)	Radimer/ Cornell: 53.0; CCHIP: 48.0; NHANES III: 17.0
Gattu, 2019, USA	XS	Examine the validity of the 2-item HVS against the 18-item HFSSM in identifying young children (< age 4) in FI households.	HVS vs. HFSSM (18-item)	Staff-administered, NFS	NS	Medical Centre - Paediatric ED and Primary Care	Caregivers (5039; 90; 84% >21 yrs)	HVS: 31.6; HFSSM: 21.5
Gundersen, 2017, USA	XS	Examine the performance of two-item FI screening questions using the USDA (6-item HFSSM) in census data.	HFSSM (6-item)	Staff-administered, NFS	NA	National Survey	Participants (60 000; NFS)	NS

Hager, 2012, USA	XS	Develop a brief screen to identify families at risk for FI and to evaluate the validity, using Children's HealthWatch data.	HVS vs. HFSSM (18-item)	Staff-administered, NFS	NS	Medical Centres - Paediatric	Patients (via caregiver) (30,098; 46; 0-36 months)	HVS: 35.0; HFSSM: 23.0
Harle, 2023, USA	XS	Assess the accuracy of eMR FI questions.	HVS vs. HFSSM (6-item)	Self-administered, NFS	Yes	Community - Health Clinics	Patients (826; 64; x̄ 50 yrs)	HVS: 38.0; HFSSM: 36.0
Harrison, 2021, USA	XS	Validate the HVS within a general medicine adult population.	HVS vs. HFSSM (18-item)	Staff-administered, digital	NS	Community - Medical Centres	Patients (295; 58; 18-93 yrs)	HVS: 24.8; HFSSM: 17.6
Kerz, 2021, Australia	XS	Identify questions suitable for screening households at risk of FI using various screening tools.	NHS vs. FAO-FIES vs. HVS (2-item) vs. HFSSM (18-item)	Self-administered, paper and pen OR digital	No	Outpatient - Paediatric	Caregivers (122; 81; 83% aged 25-49 yrs)	Proposed screener-HFSSM Q2&3: 45.1; NHS: 18.9; FAO-FIES: 45.5; HVS: 40.5; HFSSM: 41.0
Kleinman, 2007, USA	XS	Examine the use and accuracy of a brief screening tool to identify family hunger.	1 Qu (hunger - 1 mth) vs. HFSSM (18-item)	Self-administered, paper and pen	No	Community - Health Centre	Patients (via caregivers) (122; 47; x̄ 4.6 yrs)	Single Qu: 49.0; HFSSM: 46.7
Lane, 2014, USA	RCT	Evaluate the validity of a single item FI screen.	1 Qu (worry - 12 mth) vs. HFSSM (18-item)	Self-administered, paper and pen	No	Community - Paediatric Clinic	Caregivers (205; 90; x̄ 25.3 yrs)	30.0
Makelarski, 2017, USA	XS	Test the diagnostic accuracy of the AAP compared to the HVS and 6-item HFSSM.	AAP vs. HVS & HFSSM (6-item)	Self-administered, paper and pen	NS	Hospital - Adult and Paediatric ED	Patients: 12-month survey (154; NS; >18 yrs)<br>30-day survey (118; NS; >18 yrs)	30 days - AAP: 31.0; HVS: 46.0; HFSSM: 39.0<br>12 month - AAP: 39.0; HVS: 53.0; HFSSM: 46.0
McKay, 2022, Australia	XS	Determine prevalence FI among pregnant people using different FI	HVS & HFSSM (6-item) vs.	Self-administered, digital	No	Online - Survey	Respondents (303; 100; NS)	Single-item: 6.2;

		assessment tools to determine screening question combination.	HFSSM (10-item)					HVS: 11.4; HFSSM (6-item): 11.7; HFSSM (10-item): 14.3
Radandt, 2018, USA	XS	Validate a 2-item written FI screen in a dental clinic setting.	HVS vs. HFSSM (6-item)	Self-administered, paper and pen	No	Academic - Paediatric Dental Clinic	Caregivers (141; 82; 18+ yrs)	HVS: 41.1; HFSSM: 31.2
Swindle, 2013, USA	XS	Validate a 2-item screen for FI that can be integrated into early childcare settings.	EC-FM vs. HFSSM (6-item)	Staff-administered, NFS	No	Community - Childcare	Survey: Caregivers (1050; 89; NS) Validation: (53; NFS)	EC-FM: 26.4; HFSSM: 22.6
Tran, 2022, USA	XS	Validate the NutriSTEP FI question compared against HVS and the HFSS screening tools.	NutriSTEP vs. HVS & HFSSM (18-item)	Self-administered, digital	No	Community - Early Childhood Program	Caregivers (55; 89; x̄ 32 yrs)	>40 by all tools
Urke, 2014, Canada	XS	Validate a single FI question in an Inuit population by testing each question in the 18-item HFSSM.	HFSSM (18-item)	Staff-administered, NFS	No	Inuit Health Surveys	Survey respondents (2150; NFS)	NS
Vaudin, 2020, USA	XS	Examine the development and feasibility of the Expanded Food Security Screener (FSS-Exp) (expanded HFSSM 6-item) in older adults.	FSS-Exp	Staff-administered, paper and pen or digital	No	Community - Meal Delivery Service	Participants (148; NS; >60 yrs)	37.2
Vest, 2021, USA	XS	Compare estimates of social risk factors from screen questions against estimates from validated instruments.	HVS & ICD-10 Z codes vs. HFSSM (6-item)	Self-administered, digital	Yes	Medical Centre - ED and Primary Care	Patients (52; NS; >18)	HVS: 37.2; ICD-10 Z code: 2.0; HFSSM: 39.9
Young, 2009, Australia	XS	Validate a two-item FI screener in a population of adults living with HIV.	HFSSM (2-item) (food didn't last, balanced meals -	Self-administered, NFS	NS	Community - HIV clinic	Patients (49; 4; x̄ 45 yrs)	NS

			12 mth) vs. HFSSM (6-item)					
**Implementation/experience papers**								
Acquah, 2020, USA	XS	Compare FI screening strategies to determine which method would identify the largest number of patients.	HVS	Multiple	Yes	Outpatient Medical Clinic	Clinic Staff (42; NFS)	12.2
Adams, 2017, USA	Qual: Focus group	Assess the attitudes of providers to the implementation of FI screening.	HVS	Self-administered, paper and pen	Yes	Community - Academic Paediatric General Practice	Health Care Providers (NFS)	NS
Barnidge, 2017, USA	XS	Identify HP readiness to screen patients and assess the perceived barriers to conducting FI screening.	HVS	Self-administered, paper and pen	NS	Hospital - Paediatric Centre	Physicians (67, NFS) Caregivers (212; 90; median age 31 yrs)	57.1
Barnidge, 2020, USA	Qual: Interviews	Explore caregivers' barriers and facilitators to FI disclosure.	HVS	Self-administered, digital	NS	Hospital - Paediatric Centre	Caregivers (15; 87; >18)	100
Bernhardt, 2022, USA	Mixed Methods: XS; Interviews	Examine what elements of patient-centred care affect patient comfort in discussing FI screening.	2 Q, NFS	Mixed: self-administered, digital OR Staff-administered, digital	NA	Community - Clinical Organisations and Food Pantries	Patients - Survey: (46; 64, two thirds were ages 30-39); Interviews: (12; NFS)	50
Black, 2022, USA	QI	Increase screening for FI, to identify key drivers to screen patients.	HVS	Self-administered, digital	Yes	Outpatient - Paediatric Cardiology Clinic	Patients (via caregiver) (5604; 46; 20-204 months)	5.2
Burkhardt, 2012, USA	QI	Use quality-improvement methods to increase identification of household FI	HVS	Staff-administered, digital	Yes	Outpatient - Paediatric Primary Care Centre	Clinicians (24; 67; 26-35 yrs)	NS (identification rate: 11.2%)

Caldwell, 2023, USA	Mixed Methods: XS; Interviews	Characterise patients experiencing FI and contextualise patient experiences with screening for FI	HVS	Self-administered, NFS	Yes, in some clinics	Community - Health Clinics	Patients (survey) (1013; 76; 18-96 yrs)<br>Health Clinic Staff (interviews) (12; NFS)	52.8-60.7 (two questions)
Canavan, 2022, USA	Qual: Interviews	Assess barriers and facilitators prenatal care clinics face in addressing FI among pregnant people.	NA	Multiple: staff and self-administered, paper and pen or digital	NA	Outpatient - Prenatal Care	Health Care Providers (9; NFS)	N/A
Chiu, 2019, USA	QI	Assess prevalence of FI and identify barriers to implementation of screening.	HVS	Multiple	Yes	Community Health Centres	Patients (1493; NFS)	41.5
Corbera-Hincapie, 2022, USA	Qual: Interviews	Explore perceptions surrounding FI screening to improve identification and provision of resources.	NA	NA	NA	Outpatient - Cystic Fibrosis (CF); CF Support Group	Patients (via caregiver if <18 yrs) (20; 65; 2-38 yrs)	100
Cullen, 2019, USA	Randomised trial	Compare caregiver preferences for screening methods to maximise disclosure.	HVS	Mixed: staff and self-administered, digital	No	Hospital - Paediatric ED	Patients (via caregiver) (1818; NS; x̄ 10 yrs)	20.6
Cullen, 2020, USA	Qual: Interviews	Explore the effects of screening modality and location on caregivers' comfort and willingness to disclose FI	HVS	Staff-administered, digital	No	Hospital - Paediatric ED	Patients (via caregiver) (40; NS; x̄ 10 yrs)	20.6
Fleeger, 2007, USA	XS	Explore families' experiences regarding screening and referral for FI; and acceptability of screening.	CCHIP	Self-administered, digital	NS	Paediatric Clinics	Caregivers (193; 90; x̄ 29 yrs)	39.0
Frost, 2022, USA	XS	Assess HP knowledge, referral practices, and	NA	NA	NA	State-based Health System	Health Care Providers (280; NFS)	N/A

		barriers for FI policy and practice implementation.						
Gonzalez, 2021, USA	XS	Assess the prevalence of FI among emergency department patients and feasibility of screening.	HVS	Staff-administered, NFS	No	Hospital - Paediatric ED	Patients (via caregiver if <18 yrs) (439; 45; median 6 yrs)	17.5
Gore, 2022, USA	QI	Develop and implement a collaborative, sustainable process to screen hospitalised adults for FI.	HVS	Staff-administered, digital	Yes	Hospital - Adult Inpatient	Patients (361; NS; >18 yrs)	5.8
Higginbotham, 2019, USA	QI	Implement FI screening and referral process during well-child appointments.	HVS	Self-administered, NFS	NS	Rural Health Clinic	Patients (via caregiver) (53; NS; 0-5 yrs)	16.9
Hoisington, 2012, USA	XS	Determine the extent to which physicians and nurse practitioners monitor household food insecurity of families; examine factors that influence food insecurity monitoring.	NA	NA	NA	Community - General Practice	Health Practitioners (186; 74; 26-78 yrs)	N/A
Knowles, 2018, USA	Qual: Interviews; focus group	Evaluate efficacy of screening and referral process.	HVS	Mixed: staff-administered, digital OR self-administered, paper and pen	Yes	Hospital Outpatient - Paediatric	Caregivers (19; 95; 22-38 yrs) Health Practitioners (11; 82; 22-63 yrs)	15.6
Kopparapu, 2020, USA	XS	Explore patient attitudes regarding screening for FI and patient preferences for intervention.	HVS	Self-administered, paper and pen	No	Academic Hospital Primary Care Clinics	Patients (284; NS; >18 yrs)	40.8
Kuehne, 2023, UK	XS	Assess HP knowledge, skills, and views on FI.	NA	NA	NA	Community - Eating Disorder Clinic	Health Practitioners (93; 88; 18-75 yrs)	NA
Lundeen, 2017, USA	Qual: Interviews	Assess programs that screen patients (especially older adults) for FI	HVS + various	Staff-administered, NFS	Yes (Some)	Community - Health Care Programs for older adults	Health Care Programs (22; NFS)	NA

Markowitz, 2022, USA	XS	Examine the pattern of hospitals that screen for FI; characterise protocols; and describe interventions.	Various	Staff-administered, NFS	Yes	Hospital - Inpatient & ED	Hospitals (76)	NS
Nederveld, 2022, USA	Mixed Methods: XS; Interviews	Explore stakeholder perspectives on data sharing for FI screening and referral in primary care.	HVS	Staff-administered, NFS	NS	Community - Medical Practices and Community Events	Interviews: Patients & staff (42; 88; 20-79 yrs) Survey: Patients & staff (126; 69; 20-89 yrs)	38.9
Nguyen, 2023, USA	XS	Evaluate the prevalence of screening for FI during the COVID-19 pandemic using clinic encounters.	HFSSM (3-item) (worry, food didn't last, balanced meals - 12 mth)	Staff-administered, digital	Yes	Community - Health Centres	Staff-Patient Encounters (275,465; 50; all ages)	NS
Okafor, 2020, USA	Mixed Methods: XS; focus groups	Identify the challenges associated with universal screening in clinics.	HVS	Staff-administered, digital	Yes	Community - Private Paediatric and Family Medicine Clinic	Survey: Patients (1299; 58; all ages) Focus Group: Health Practitioners (NFS)	42.0
Orr, 2019, USA	Qual: Interviews	Explore caregiver experience, acceptability of clinic-based screening, and effectiveness of referral.	HVS	Self-administered, paper and pen	NS	Community - Academic Paediatric Clinic	Caregivers (17; 100; x̄ 33 yrs)	100
Palakshappa, 2017a, USA	Qual: Interviews	Determine caregivers' perceptions of FI screening in suburban paediatric practices.	AAP	Staff-administered, digital	Yes	Community - Paediatric Medical Practice	Caregivers (23; 100; x̄ 31 yrs)	100
Palakshappa, 2017b, USA	Mixed Methods: XS; focus groups	Evaluate clinicians' views on the feasibility, acceptability, and impact of FI screening in suburban practices.	AAP	Staff-administered, digital	Yes	Community - Paediatric Medical Practice	Survey: Patients (via caregiver) (4,371; 49; x̄ 17-20 months) Focus groups:	2.8

							Clinicians (18; 83; NS)	
Palakshappa, 2020, USA	XS	Determine the difference in FI disclosure rates by parents/guardians screened by a written questionnaire compared to verbally by the clinician.	HVS	Mixed: self-administered, paper and pen vs. Staff-administered, digital	Yes	Hospital Outpatient - Paediatric Clinic	Patients (via Caregiver) (7,996; NS; <18 yrs)	14.3
Pooler, 2018, USA	Qual: Interviews	Explore HP attitudes, knowledge, beliefs and perceived barriers about implementing FI screening for patients > 50 yrs.	NA	NA	NA	Primary Care Providers	Primary Care Providers (16; 87; NS)	NA
Smith, 2017, USA	Intervention trial	Determine if a FI education intervention modifies knowledge, attitudes, and clinical behaviour.	HVS & HFSSM (6-item)	Staff-administered, digital	Yes	Academic - Medical School	Health providers (85; NFS)	NS
Stenmark, 2018, USA	QI	Evaluate barriers from the implementation of FI screening and referral.	HVS	Self-administered, paper and pen	NS	Community - Paediatric Clinics	Two clinics	12-18 (two sites)
Taher, 2022, USA	Qual: Interviews	Understand implementation processes and outcomes of two different FI screening initiatives in primary care.	HVS	Staff-administered, NFS	Yes	Government Healthcare & Private Medical Centre	Healthcare staff (19; NFS) Patients (1576; NFS)	27.9 (average across sites)
Thomas, 2018, Canada	Qual: Interviews; focus group	Explore the acceptability and feasibility of FI screening in diabetes care.	HFSSM (3-item) (worry, food didn't last, skip meals - 3 mth)	Staff-administered, digital	Yes	Hospital Outpatient - Diabetes	Survey: Patients (33; NS; >18 yrs) Interviews: Patients (14; 64; 48-82 yrs) Focus Group: Care providers P1: (15; NFS)	39.0

Vitale, 2019, Canada	Mixed Methods XS; Interviews	Assess the acceptability and feasibility of FI screening; identify perceived facilitators and barriers.	HFSSM (3-item) (worry, food didn't last, skip meals - 3 mth)	Staff-administered, NFS	NS	Outpatient - Paediatric Diabetes	Screening: Caregivers (37; 84; x̄ 44 yrs) Interviews: Health Professionals (3; NFS)	40.5

NA = Not applicable; NFS = No further specification; NS = Not specified
XS= Cross-sectional; RCT = randomised controlled trial; QI = Quality improvement
HP = Health Professional; FI = Food Insecurity
ED = Emergency Department; eMR= Electronic Medical/Health Record

USDA = United States Department of Agriculture

AAP = American Academy of Pediatrics
CCHIP = Childhood Community Hunger Identification Project (8-Item)
EC-FM = The Early Childhood Family Map Inventory (2-Item from HFSSM)
FAO-FIES = Food and Agricultural Organisation Food Insecurity Experience Scale (8-Item)
FSS-Exp = Expanded Food Security Screener (10-Item expanded version of HFSSM 6-Item)
HFSSM (3-Item) = USDA Household Food Security Survey Module (3-Item)
HFSSM (6-Item) = USDA Household Food Security Survey Module: 6-Item Short Form
HFFSM (18-Item) = USDA Household Food Security Survey Module: 18-Item
HVS = Hunger Vital Sign™ (Hager) (2-Item)
NHANES III = Third National Health and Nutrition Examination Survey
NHS = Australian National Health Survey (1-Item)
NutriSTEP = Nutrition Screening Tool for Every Preschooler (1-Item)
